# In situ donor keratometry in deceased patients as a novel screening technique for eye banking

**DOI:** 10.1007/s00417-022-05871-8

**Published:** 2023-01-11

**Authors:** Adrien Quintin, Loïc Hamon, Achim Langenbucher, Berthold Seitz

**Affiliations:** 1 Department of Ophthalmology, Saarland University Medical Centre (UKS), Kirrberger Straße 100, Bld. 22, 66424 Homburg, Saar, Germany; 2 Institute of Experimental Ophthalmology, Saarland University, Homburg, Saar, Germany

**Keywords:** Keratometry, Optical coherence tomography, Donor cornea—Eye banking, Corneal transplantation, Keratoplasty

## Abstract

**Purpose:**

To investigate the potential role of keratometry on whole globes in situ of deceased patients by assessing its repeatability and comparing it with sterile donor tomography after excision and preservation in organ culture.

**Methods:**

A sequence of 5 measurements was taken from 40 eyes in situ of deceased patients < 24 h after death using the portable Retinomax K-plus 3 (Bon, Tokyo, Japan). Keratometry of whole globes in situ, from which sclerocorneal discs were taken for organ culture, was compared to those obtained after measuring these sclerocorneal disks through their cell culture flask in medium I after 5 ± 4 days using the anterior segment optical coherence tomograph Casia 2 (Tomey Corp., Nagoya, Japan), and to 964 different donor corneas in medium II.

**Results:**

Cronbach’s alpha of the in situ keratometry was 0.891 and 0.942 for the steepest and flattest corneal power (*P*). The steepest (44.5D) and flattest (41.1D) *P* as well as the astigmatism (3.4D) of in situ corneas remained unchanged after preserving sclerocorneal discs in medium I (respectively 44.7D [*p* = 0.09]; 41.4D [*p* = 0.17]; 3.3D [*p* = 0.09]). The comparison of the in situ values with the 964 measured different donor corneas in medium II showed significantly (*p* < 0.001) higher *P* at the steep (45.4D) and flat (43.9D) meridian and smaller astigmatism (1.4D) for sterile donor tomography.

**Conclusions:**

Measuring deceased patients’ eyes in situ with the portable Retinomax K-plus 3 represents a feasible and reliably repeatable screening method in the eye bank. In comparison to donor tomography in medium I, it measures a similar power and astigmatism.


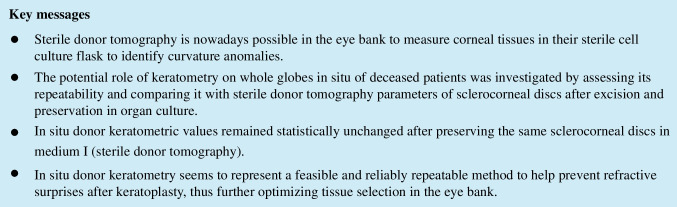


## Introduction

Any corneal microsurgeon who performs high-volume penetrating keratoplasties (PKP) may have inadvertently transplanted donor corneas with (subclinical) keratoconus or with a history of keratorefractive surgery, which typically entails significant postoperative refractive disadvantages for the patient [1–4]. Such pre-existing pathologies and conditions after keratorefractive surgery, the latter of which are increasingly common in donor tissues, cannot be reliably recognized in all cases by visual inspection at the slit lamp alone. Therefore, several authors have emphasized the need for improved screening of donor tissues to better detect corneas with refractive anomalies such as keratoconus or with prior refractive procedures such as laser-assisted in situ keratomileusis (LASIK), photorefractive keratectomy (PRK), or small incision lenticule extraction (SMILE) preoperatively in the eye bank [5–8]. Previous studies showed a false-negative rate of 3.4–50.0% for the identification of post-LASIK donor corneas, depending on whether detection was based solely on slit lamp examination and clinical history or a combination of both [9–11]. Given the increasing number of performed refractive procedures worldwide, eye banks will increasingly face the problem of how to identify corneal donor tissues of such pre-operated eyes.

In this context, many authors have focused on the potential role of donor tomography in the eye bank. Initial studies on this subject were already performed in 2003 by Priglinger et al. [12] on a time-domain optical coherence tomography (OCT), which was originally designed for retinal imaging. In 2007, Lin et al. [13] introduced a method to identify prior LASIK in donor corneas by measuring the anterior curvature and anterior/posterior stromal reflectivity ratio with OCT scans. The use of OCT as a screening method has been improved by Janunts et al. [14], who presented the application of donor tomography for a swept-source anterior segment optical coherence tomograph (AS-OCT), although the examined corneas needed to be inserted in a special examination container for this purpose. Finally, Damian et al. [15] presented the use of a spectral-domain OCT.

Despite the scientific advances, sterile measurement of sclerocorneal disks in a sterile container has been a serious challenge for a long time due to the flat interfaces of this latter. Nevertheless, our group [14–16] succeeded in enabling and approving a new concept to measure donor corneas tomographically in their sterile cell culture flask in order to identify curvature anomalies (high astigmatism, keratoconus, prior keratorefractive surgery). For the first time, donor tomography was possible without requiring a separate examination container, enabling sterile conditions during measurements.

In addition to the development of donor tomoraphy, other methods have been proposed throughout the years as refractive screening devices in the eye bank. Stoiber et al. [7] presented Placido disk videokeratography, which could detect curvature deviation of the anterior corneal surface, as an alternative approach to detect curvature anomalies in donors. This method requires an artificial increase in intraocular pressure for a reliable topographical reading and is therefore limited to whole globes rather than sclerocorneal discs. Terry et al. [8] examined enucleated globes using Scheimpflug tomography, which allowed measurement of keratometry and pachymetry of donor eyes. Both of the abovementioned techniques [7, 8] represent whole globe examinations. However, in most eye banks in Europe, sclerocorneal discs are harvested instead of whole globes, which renders an examination with these methods [7, 8] not easily applicable in all eye banks.

The purpose of this study was to examine a potential new screening technique to avoid postoperative refractive surprises after corneal transplantation, as an alternative for sterile donor tomography for eye banks lacking an AS-OCT. In this context, the potential role of keratometry on whole globes in situ of deceased patients was investigated by assessing its repeatability and comparing it with AS-OCT parameters of sclerocorneal discs after excision and preservation in organ culture.

## Methods

Firstly, consent for corneal harvesting was obtained on the basis of an organ donor card (according to the law on donation, removal and transfer of organs and tissues “TPG” [“deutschen Transplantationsgesetz”] § 3) or from relatives (according to TPG § 4). Subsequently, a detailed medical and surgical history was collected from the donor’s medical records to exclude contraindications based on the guidelines of the European Eye Bank Association (EEBA) [17], whereafter the potential donor corneal tissues were considered suitable for explantation.

### In situ donor keratometry

Prior to corneal explantation, 44 eyes from deceased patients were measured in situ using the portable Retinomax K-plus 3 (Bon, Tokyo, Japan) within an interval of 1 day after death. First, a Mellinger eye speculum (Bausch & Lomb GmbH, Heidelberg, Germany) was used to keep the eyelids apart. Then, a drop of sodium hyaluronate (0.2%) was instillated to ensure a more regular corneal surface before keratometric reading. In situ measurements with the portable Retinomax K-plus 3 were repated 5 times consecutively.

### Corneal harvesting and storage

Donor corneal tissues were harvested in a sterile manner as sclerocorneal disks with a diameter of 15 mm. After harvesting, the donor corneas were preserved in our Klaus Faber Centre for corneal diseases incl. LIONS Eye Bank Saar-Lor-Lux, Trier/Westpfalz in Homburg/Saar. The sclerocorneal disks were stored in cell culture flasks (Primaria 25 cm^2^ Canted-Neck Cell Culture Flask, Corning Inc., Corning, NY, USA) that were initially filled with an isotonic (307 mOsmol/kg) nutrient medium (medium I) (Biochrom AG, Berlin, Germany). This standard organ culture medium I (pH scale 7.2–7.3) is composed of 10% Minimum Essential Medium (MEM) with Earle’s balanced salts supplemented with 1% penicillin/streptomycin, 1% amphotericin B (250 μg/mL), 1% l-glutamine (200 mM), 1.25% Hepes buffer (1 M), 2.93% NaHCO3, and 2% fetal calf serum. Due to the absence of dehydrating agents, the donor corneas swell considerably in this medium. The tissues were stored in this organ culture at 34 °C for a maximum of 34 days. Donor corneas designated for penetrating keratoplasty based on tissue evaluation (endothelial cell count and morphology, microbiology, slit lamp examination according to § 20c of the German medicine law for medical products “AMG” [“Arzneimittelgesetz”]) were transferred 1–3 days prior to surgery into another culture medium (medium II) (Biochrom AG, Berlin, Germany) in the eye bank’s laminar flow unit. This hypertonic medium (353 mOsmol/kg) contains 6% dextran T500, a hydrophilic macromolecule that induces colloid osmotic pressure and thus deswells the corneas in order to obtain a corneal graft thickness comparable to that of the recipient cornea at the time of surgery.

### Sterile donor tomography

**Fig. 1:**
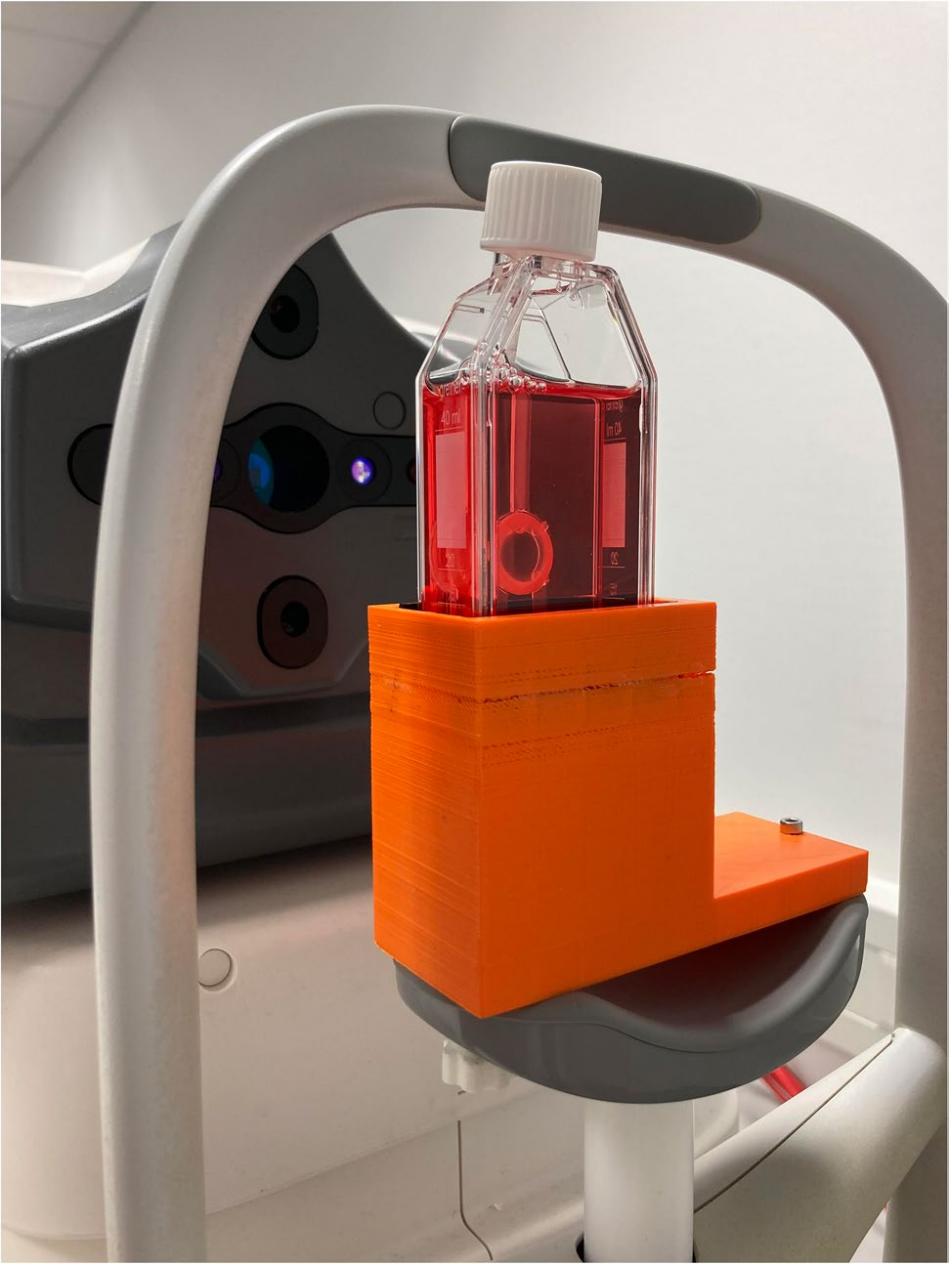
**Sterile donor tomography**. Preoperative measurements of donor corneal tissues were conducted using the anterior segment optical coherence tomograph (AS-OCT) in a sterile way through their cell culture flask, which was mounted on the chin rest of the AS-OCT in a holder previously constructed using a 3D printer

Measurements of 964 sclerocorneal disks were routinely performed using the AS-OCT CASIA 2 (Tomey Corp., Nagoya, Japan). These preoperative donor tomographies were carried out at least 12 h after transfer of the corneal tissues into medium II in order to enable a reliable measurement of the donor corneal tissue after deswelling [18]. This OCT technique reaches a lateral measurement range of about 7-mm diameter, mainly limited by the tissue holder, and a depth range of up to 13 mm, enabling a complete recording of the donor corneas within their culture flasks [16]. Sterile- and contact-free measurements of the donor corneas could therefore be performed through their sealed cell culture flask, which was placed on the chin rest of the AS-OCT in a 3D-printed holder (Ultimaker 2 Go, Ultimaker B.V., Geldermalsen, The Netherlands) (Fig. 1). A 3D volume data set was generated, and the measured raw data were imported into MATLAB (MathWorks Inc., Natick, Massachusetts, USA) and analyzed. After preprocessing the images to remove artifacts that may occur due to the flask wall and cornea holder, edge detection of the anterior and posterior corneal surface was applied according to the method of Mäurer et al. [15, 16]. Finally, the refractive power of the corneal front and back surface in the steep and flat meridian, as well as the central corneal thickness (measured at the apex) were determined.

All the means (*n* = 44) of the repeated in situ keratometric readings were compared to the routinely measured sterile tomography of 964 donor corneas using a Mann–Whitney *U* test in regards to the dioptric power (*P*) at the steep and flat meridian of the cornea, as well as the corneal astigmatism. The respective keratometric values of the whole globes, from which sclerocorneal discs were harvested for organ culture in the eye bank, were also compared to those obtained after measuring the same sclerocorneal discs in medium I (*n* = 25) after 5 ± 4 days, using a Wilcoxon test.

A statistical analysis of the above mentioned parameters was carried out using SPSS (IBM Corp., NY, USA) version 20. Result values are expressed as mean x̄ ± standard deviation (SD) (minimum–maximum) unless otherwise stated. A *p*-value of < 0.05 was considered statistically significant.

## Results

The mean standard deviation of the five in situ measurements (*n* = 40) was 1.3 D and 0.9 D for the power (*P*) at the steep and flat meridian of the cornea, respectively, and 1.4 D for keratometric astigmatism. The corresponding Cronbach’s alphas were 0.891, 0.942, and 0.794, respectively.

**Fig. 2:**
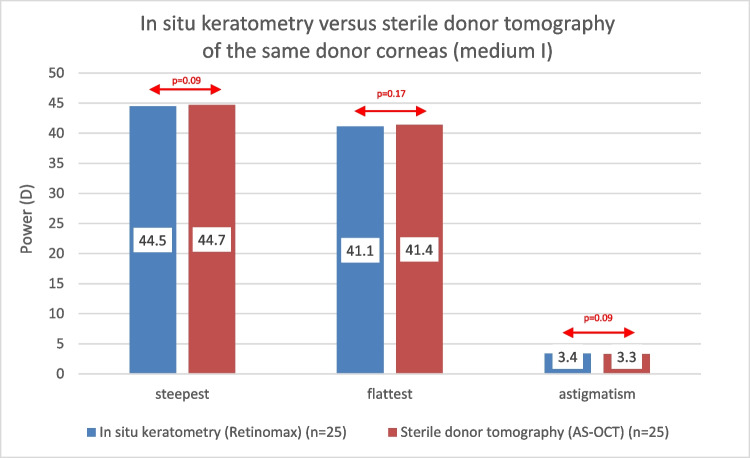
**Comparison of in situ keratometry in deceased patients with sterile tomography in organ culture medium I of the same donor corneas after explantation**. The steepest and flattest power and the astigmatism of the donor corneas did not differ statistically significantly when measured with in situ keratometry measured with the Retinomax in comparison to sterile tomography using an anterior segment optical coherence tomograph (AS-OCT) after explantation and preservation in organ culture medium I (p-values respectively 0.09, 0.17, 0.09). A red arrow refers to a non statistically significant difference

The steepest *P* (44.5 ± 2.3 D) of in situ corneas remained unchanged (*p* = 0.09) after preserving the same sclerocorneal discs (*n* = 25) in medium I (sterile tomography 44.7 D ± 2.1 D) after 5 ± 4 days. Again, the flattest *P* (41.1 ± 1.8 D) of in situ corneas remained unchanged as well (*p* = 0.17) compared to sterile tomography of the same sclerocorneal discs after explantation (41.4 ± 2.0 D). The keratometric astigmatism (3.4 ± 1.8 D) also remained unchanged (*p* = 0.09) in comparison to tomographic values in medium I (3.3 ± 1.8 D) (Fig. 2).

**Fig. 3:**
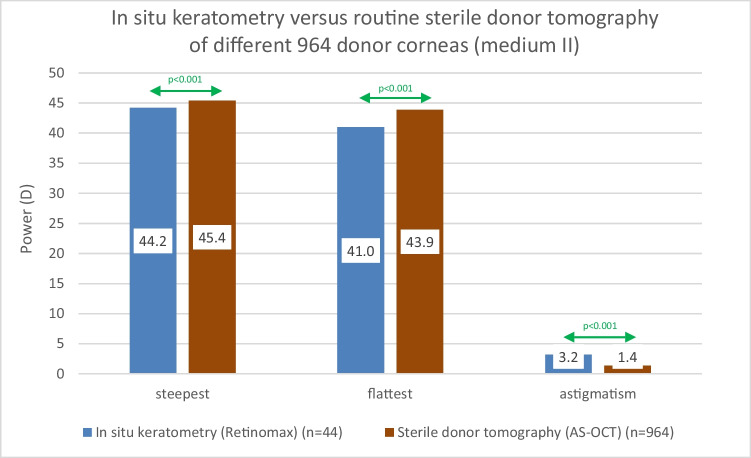
**Comparison of in situ keratometry in deceased patients with routinely in organ culture medium II measured sterile tomography of different 964 donor corneas**. Both the in situ keratometric steepest and flattest power as well as the keratometric astigmatism of donor corneas measured with the Retinomax differed statistically significantly (p<0.001) from the values measured with ster th ste ile tomography using an anterior segment optical coherence tomograph (AS-OCT) after explantation and preservation in organ culture medium II. A green refers to means a statistically significant difference

However, the comparison of the keratometry of in situ corneas (*n* = 44) of deceased patients with the routinely measured sterile tomography of 964 different donor corneas in medium II showed a statistically significantly different (*p* < 0.001) value for all examined parameters. Indeed, *P* at the steep meridian was measured significantly (*p* < 0.001) smaller (i.e., flatter) with in situ keratometry (44.2 ± 2.5 D) in comparison to sterile tomography (45.4 ± 1.8 D). Again, *P* at the flat corneal meridian was measured significantly (*p* < 0.001) smaller (i.e., flatter) with in situ keratometry (41.0 ± 1.8 D) in comparison to sterile tomography (43.9 ± 1.3 D). The keratometric astigmatism (3.2 ± 2.0 D) was significantly (*p* < 0.001) higher than the sterile tomographic astigmatism (1.4 ± 1.7 D) (Fig. 3).

Those results, compared to the standard values of the corneal determinants according Gullstrand’s model eye, are displayed in Table 1. Furthermore, results of the donor tomography are presented in detail together with the corresponding value at the steep and flat meridian of the anterior and posterior corneal surface. The refractive power at the steep/flat meridians of the anterior surface of the donor corneas was 50.5 ± 2.0 (45.4–62.0) D/48.9 ± 1.5 (42.7–56.1) D, while the corresponding values for the posterior corneal surface were − 6.2 ± 0.3 (− 7.5 to − 5.3) D/ − 5.9 ± 0.3 (− 6.9 to − 5.0) D. The mean central corneal thickness was 612.4 ± 82.1 (378.5–1029.0) μm.

**Table 1:** Comparison of in situ keratometry, sterile donor tomography, and reference of the Gullstrand normal eye. Result values are expressed with mean ± standard deviation (minimum – maximum) [median]. In contrast to donor tomography, in situ donor keratometry does not allow further analysis of the posterior curvature nor corneal thickness

			Gullstrand eye model	In situ keratometry (*n* = 44)	Sterile donor tomography (*n* = 964)
Refractive power (D)	Anterior surface	Steep	48.8		50.5 ± 2.0 (45.4–62.0) [50.0]
		Flat			48.9 ± 1.5 (42.7–56.1) [48.9]
	Posterior surface	Steep	-5.9		− 6.2 ± 0.3 (− 7.5 to − 5.3) [− 6.1]
		Flat			− 5.9 ± 0.3 (− 6.9 to − 5.0) [− 5.9]
	Total	Steep	43	44.2 ± 2.5 (40.1–50.8) [44.0]	45.4 ± 1.8 (40.8–55.6) [44.8]
		Flat		41.0 ± 1.8 (37.6–45.3) [40.8]	43.9 ± 1.3 (38.3–50.4) [43.9]
Central corneal thickness (μm)			500		612 ± 82 (379–1029) [598]

## Discussion

Both methods under test allow for a sterile measurement method for eye banking. Indeed, donor tomography permits sterile, direct, and non-contact tomographic imaging of corneal donor tissue within their cell culture flasks [14–16], without risk of contamination of donor tissue. In situ donor keratometry also allows for a sterile measurement (non-contact method) of donor corneas prior to their harvesting.

The method of sterile donor tomography, which has been routinely performed in Homburg/Saar since 2018, takes approximately 12 min for each cornea (2 min for measurement and 10 min for evaluation) [19]. On the contrary, in situ donor keratometry only takes about 15 s, i.e., a clearly shorter duration than its tomographic counterpart.

However, in contrast to donor tomography, in situ donor keratometry does not allow further analysis of the posterior curvature nor corneal thickness. In this context, the sterile donor tomography allows a more detailed analysis of donor corneas in the eye bank. Moreover, donor tomography allows the detection of corneal tissues with *morphological* (scars, corneal dystrophies) as well as curvature anomalies (high astigmatism, keratoconus, or prior keratorefractive surgery) [14–17], whereas in situ donor keratometry only enables the detection of keratorefractive anomalies.

In contrast to in situ donor keratometry, uneven epithelial coating between corneas in organ culture could lead to differences in donor tomographic measurements. Indeed, Neubauer et al. [20] showed that the epithelium slightly thickens after 2 to 3 days in culture medium I, after which epithelial loss can be observed, followed by a complete reepithelialization within 7 to 10 days. As the measurements were not analyzed with regard to the epithelial layer thickness, this variable could slightly change the sterile donor tomographic results. Despite its (thought to be) unaffected epithelial coating, in situ donor keratometry is however highly sensitive to the quality of the precorneal tear film quality for obtaining valid keratometric reading [21]. A drop of sodium hyaluronate has to be instillated to ensure a more regular corneal surface, without which the quality of the keratometric reading could be compromised. However, it has to be mentioned that this substitution with artificial tears is undoubtedly paired with measurement’s artifacts as it may considerably influence the corneal topography.

During in situ donor keratometry, the corneas were examined in their natural environment. However, pressure applied by the eye speculum (eyelid retractor) could still have biased the measured radii of curvature by distortion following mechanical stress. During sterile donor tomography, sclerocorneal disks are examined in their cell culture flask. The storage and attachment of these corneas to the cell culture flask holder may cause slight deformation of the donor corneal tissue, rendering the measured geometry inconsistent with in situ conditions [19, 22, 23].

Independent of its clinical relevance, in situ keratometry seems to represent a highly repeatable measurement method with regard to the calculated Cronbach’s alpha [24]. Sterile donor tomography has also been proven to be highly reliable for the assessment of the corneal thickness with both a manual and the (routinely used) semi-automated method [25]. The repeatabilty of the curvature measurement with sterile donor tomography is currently being investigated in our eye bank.

With regard to the aforementioned results presented in this study and regardless of the above-described (dis)advantages of in situ keratometry, this technique on whole globes of deceased patients seems to represent a suitable screening method for eye banking when compared to sterile donor tomography, as the keratometric values (admittedly from a small sample size) remained statistically unchanged after preserving the same sclerocorneal discs in medium I. Nevertheless, those values differed statistically when being compared to the routinely in organ culture medium II measured sterile tomography of 964 different donor corneas (much larger sample size). However, it has contradictorily also been shown that the (hourly measured) corneal curvature did not change within 24 h after deswelling in medium II [18], which could suggest a stable corneal curvature after tissue conversion from medium I to medium II. Ideally, a larger number of in situ corneas of deceased patients should be keratometrically measured and then compared to sterile donor tomography of the same corneas in organ culture medium II for a better statement about the suitability of the presented method.

Moreover, the validity of the donor tomographic measurements should be examined by means of available tomographic measurements during the donor’s lifetime or alternatively using histological reprocessing of unused corneal tissues to detect corneal pathologies or situations with a history of LASIK or keratokonus in respectively remarkably flat and steep donor corneas. Only then, the significance of donor tomography could be proven and be seen as a reference method with which other techniques may be compared. These projects are currently being researched in our eye bank.

Given the results and information presented in this study, the question of the clinical role of in situ keratometry arises. Much more than an alternative for eye banks lacking an AS-OCT, in situ keratometry can be considered as an “addition to” rather than as a “replacement for” sterile donor tomography. Indeed, sterile donor tomography provides a more complete screening of donor corneas, but in situ donor keratometry allows a pre-harvesting screening, avoiding the collection of extremely curved/flat corneas und thus possibly screening out likely unsuitable corneas before harvesting.

To avoid postoperative refractive surprises, donor corneas with an out of range refractive power should be discarded from penetrating (PKP) or deep anterior lamellar keratoplasty (DALK), but may potentially be suitable for posterior lamellar keratoplasty such as Descemet membrane endothelial keratoplasty (DMEK, 98.3% in 2020 in Germany [26]) or Descemet stripping automated endothelial keratoplasty (DSAEK). In Homburg/Saar, we have so far settled on tomographic curvature anomalies beyond x̄ ± 3 SD (eminence based) as being a relative contraindication for PKP or DALK [19, 23]. Similar cutoffs for keratometric values might make sense, although a larger data base would first be needed before excluding corneal tissues on the basis of their deviating refraction from a small group mean value (for comparison: already 964 sterile donor tomographies performed in Homburg/Saar versus only 44 in situ donor keratometries as part of the current experimental study).

## Conclusion

In situ donor keratometry represent a suitable, sterile, and reliably repeatable method to help prevent refractive surprises after keratoplasty, thus further optimizing tissue selection in the eye bank. Further studies about the quality of in situ keratometry should determine if this latter may represent a valid alternative for sterile donor tomography for routine screening of donor corneas in daily practice. Positive controls of donor corneas with ectasia or prior keratorefractive surgery still need to be screened prior harvesting in order to demonstrate the sensitivity of the technique in such detection.

## Data Availability

Data and material were provided and collected in the Department of Ophthalmology, Saarland University Medical Center (UKS), Homburg/Saar, Germany. Data can be made available after publication on reasonable request.
